# 2D single crystal Bragg-dip mapping by time-of-flight energy-resolved neutron imaging on IMAT@ISIS

**DOI:** 10.1038/s41598-020-77572-3

**Published:** 2020-11-27

**Authors:** Joel Strickland, Karl Tassenberg, Gareth Sheppard, Bogdan Nenchev, Sam Perry, Jun Li, Hongbiao Dong, Genoveva Burca, Joe Kelleher, Steve Irwin

**Affiliations:** 1 School of Engineering, University of Leicester, Leicester, LE1 7RH UK; 2 ISIS Pulsed Neutron and Muon Source, Rutherford Appleton Laboratory, Harwell Campus, Didcot, OX11 0QX UK; 3 Department of Mathematics, Faculty of Science and Engineering, The University of Manchester, Alan Turing Building, Oxford Road, Manchester, M13 9PL UK; 4 Rolls-Royce Plc, Po. Box 31, Derby, DE24 8BJ UK

**Keywords:** Materials science, Particle physics, Aerospace engineering

## Abstract

The cold neutron imaging and diffraction instrument IMAT, at the second target station of the pulsed neutron and muon source ISIS, is used to investigate bulk mosaicity within as-cast single crystal CMSX-4 and CMSX-10 Ni-base superalloys. Within this study, neutron transmission spectrum is recorded by each pixel within the microchannel plate image detector. The movement of the lowest transmission wavelength within a specified Bragg-dip for each pixel is tracked. The resultant Bragg-dip shifting has enabled crystallographic orientation mapping of bulk single crystal specimens with good spatial resolution. The total acquisition time required to collect sufficient statistics for each test is ~ 3 h. In this work, the influence of a change in bulk solidification conditions on the variation in single crystal mosaicity was investigated. Misorientation of the (001) crystallographic plane has been visualised and a new spiral twisting solidification phenomena observed. This proof of concept work establishes time-of-flight energy-resolved neutron imaging as a fundamental characterisation tool for understanding and visualising mosaicity within metallic single crystals and provides the foundation for post-mortem deduction of the shape of the solid/liquid isotherm.

## Introduction

**Figure 1:**
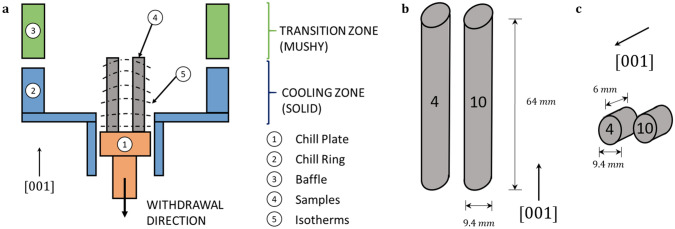
Sample preparation and geometry. (**a**) Schematic of a Bridgman furnace utilised in the manufacture of single crystal casts. The casts are withdrawn downwards through an axial thermal gradient and solidification occurs during the transition zone. The solidification front is always perpendicular to the heat flow direction. The isotherm curvature increases with distance from the chill plate. An illustration of the (**b**) bar and (**c**) disc sample geometries used in the time-of-flight energy-resolved neutron imaging experiment. CMSX-4 is labelled with ‘4′ and CMSX-10 labelled with ‘10′. The arrangement in (**b**) and (**c**) corresponds to the sample positioning in front of the MCP detector (see Fig. 9 in the method section).

Single crystal Ni-base superalloys are employed in gas turbine engines to improve high temperature mechanical properties^1,2^. They are manufactured by the Bridgman casting process^3^, which constrains the heat flow to one direction (Fig. 1a). The purpose of single crystal solidification is to eliminate detrimental grain boundaries that limit the creep ductility of the component, whilst orienting the (001) crystal structure parallel to the maximum load direction ^4^. However, the manufacture of single crystals of high quality and of practical size is no trivial task. All single crystals used in aerospace applications share the phenomenon of mosaicity ^5^, whereby individual crystalline regions within the as-cast microstructure are slightly misorientated in respect to one another. Typically, the range of bulk (001) crystallographic plane misorientation in respect to the loading direction is between 0°–15°, with above 9° being especially detrimental to the mechanical properties ^6^. Axial misorientations are undesirable, as they increase cyclic stresses during thermal cycling and degrade the high temperature performance of the material ^7^. The origin of single crystal mosaicity is thought to be related to plastic deformation during solidification ^4,5^. However, owing to the difficulties in experimental observation, the mechanism behind mosaicity is poorly understood ^8^.

In directional solidification, it has been established that the shape of the solid/liquid isotherm influences the growth direction of columnar grains ^9^, relative to the symmetry axis of the casting. The isotherm can be concave, flat, or convex, and depends on the withdrawal velocity and height from the chill plate ^10^. A concave isotherm increases the tendency for columnar grains to grow towards the middle of a casting, whereas a convex isotherm induces the grains to grow towards the external part ^10^. Consequently, any isotherm curvature must result in (001) plane misorientation and a reduction in single crystal mechanical properties. In directional solidification, the axial component of thermal gradient is the strongest at the bottom of a cast ^11^, resulting in the flattest isotherm (Fig. 1a). As a mould and solidification interface is withdrawn out of the furnace into the cooling chamber, the axial thermal gradient decreases and the isotherm curvature increases ^12^. Consequently, bulk mosaicity is expected to increase with height from the chill plate. Furthermore, the variation in (001) misorientation perpendicular to the withdrawal direction should be intrinsically related to the shape of the solid/liquid isotherm ^11^. Unfortunately, standard microstructural investigation tools, such as optical and electron microscopy, are limited to 2D sample surface analysis ^13,14^. There are some techniques for 3D reconstruction, however, they are time and labour intensive and provide only local reconstructions of small volumes ^15,16^. Consequently, these methods elucidate little information regarding the influence of bulk variations in the solidification environment on resultant (001) misorientation ^17^. Synchrotron topography studies have directly observed metallic deformation during solidification ^18,19^. However, low X-ray penetration depth within metals has restricted investigation to samples with volumes orders of magnitude smaller than typical single crystal turbine blades. Currently, no method exists which can non-destructively visualise the variation in mosaicity within bulk single crystal metallic alloys or relate (001) misorientation to changes in the shape of solid/liquid isotherm. Thus, the relationship between bulk plastic deformation during solidification, mosaicity formation, and isotherm curvature remains unknown.

Over the last 20 years the improvement in computational power and detector technology has provided a revived interest in neutron imaging ^20^. Neutrons have shown a wide range of industrial applications including use in solar cells for space travel ^21^, superconductors ^22^, and lithium batteries ^23^. They are especially good at probing objects composed of heavier elements and can penetrate thick component sections providing a better representative of bulk material characteristics ^24^. Recently, a new cold neutron imaging and diffraction instrument, IMAT, has been installed on the ISIS neutron spallation source at the Rutherford Appleton Laboratory in Oxford, United Kingdom ^25,26^. IMAT enhances the existing engineering materials analysis facilities at ISIS by adding energy-resolved neutron imaging technique options to the user programme. On the IMAT instrument, neutron energies are deduced from their time-of-flight (TOF) from the source to the TOF imaging detector and transmission spectrum is obtained for each pixel ^30^. The recorded transmission profiles are the percentage of the neutron beam that is not absorbed or scattered by a sample and represents an average over the total thickness of the specimen. The scattering component of attenuation can be separated into elastic and inelastic as well as coherent and incoherent contributions. For crystalline materials, the coherent elastic scattering part (Bragg scattering) displays a significant energy dependence in the range of cold/thermal neutrons, as wavelengths match inter-atomic distances ^27^. The principle for Bragg scattering is described through Bragg’s law:

1 $$n\lambda_{hkl} = 2d_{hkl} sin\theta_{hkl} ,$$

where, $\lambda_{hkl}$ is the wavelength of the incident radiation; $d_{hkl}$ is the crystal lattice spacing distance between lattice planes $\left( {hkl} \right)$; $\theta_{hkl}$ is the angle between the incident beam direction and the crystalline plane $\left( {hkl} \right)$; *n* is the order of reflection. For every $\lambda_{hkl}$ that fulfils the Bragg condition for a certain ($hkl$) plane, a distinct edge (for poly-crystalline materials) or dip (for single-crystalline materials) is observed in the transmission spectrum ^28^. Bragg scattering enables a change in lattice spacing from an unstrained reference to be used as a compositional or structural (phase, strain, and texture) gauge. Consequently, bulk crystallographic information can be attained from the transmission spectra and 2D ^29,30^ and 3D ^24,31^ maps created from the data. If the reader would like a broader perspective on energy-resolved imaging and neutron diffraction, please see ref ^32^.

In this work, TOF energy-resolved neutron imaging ^33,34^ is used on the IMAT beamline to investigate mosaicity within single-crystalline materials ^28^. The novel Bragg-dip mapping methodology developed within, successfully resolves variations in mosaicity within bulk single crystal microstructure and reveals new insights regarding its formation. This work has demonstrated TOF energy-resolved neutron imaging as a fundamental non-destructive single crystal characterisation tool and has provided the foundation for post-mortem deduction of the shape of the solid/liquid isotherm.

## Experimental

### TOF imaging setup on the IMAT instrument

Energy-resolved imaging is made possible by the TOF principle, where polychromatic pulses of neutrons are delivered to the beamline with a repetition rate, *f*, of 10 Hz. Once created at the spallation target, the neutrons pass through an 18 K coupled liquid hydrogen moderator that slows them to wavelengths up to 14 Å, suitable for imaging applications. The neutrons travel 42.8 m from the moderator down a supermirror guide to a pinhole selector in order to create a well-collimated beam. From the pinhole, the neutrons travel a further 10 m to a sample, which is positioned in front of the imaging detector. In this experiment, a microchannel plate (MCP) detector ^35,36^ with a pixel size of 55 μm was used to record neutron statistics. The MCP detector consists of a $2 \times 2$ array of Timepix readout ASICs, contains a $512 \times 512$ pixel matrix, and provides a $28 \times 28 \,{\mathrm{mm}}^{2}$ field of view. For each pixel, the neutron TOF relative to the trigger was measured and a transmission spectrum recorded. The wavelengths, $\lambda$, of detected neutrons were calculated from their TOF by:

2 $$\lambda = \frac{hT}{{mL_{s} }}$$

where, $h$ is Planck’s constant; $T$ is the neutron TOF; $m$ is the neutron mass;$L_{s}$ is the flight path from source to detector. The advantage of the TOF method is that each pulse contains the full neutron spectrum and so a large number of energy-dependant features (Bragg-dips) can be observed in one acquisition. On IMAT, $L_{s} = 56\,{\mathrm{m}}$ and $f = 10\, {\mathrm{Hz}}$, which provides a non-overlapping neutron bandwidth of ~ 7 Å. For a more detailed overview of the TOF imaging set-up on IMAT the reader is referred to ref ^37^.

### Sample description

For this study, CMSX-4 and CMSX-10 single crystal Ni-base superalloys samples were manufactured by Rolls-Royce Plc via the Bridgman casting process, schematic illustrated in Fig. 1a. The samples were solidified in a cluster mould arrangement under an axial thermal gradient ranging between 10–20 K/cm and kept in their as-cast (non-solutionised) state. CMSX-4 is a second-generation superalloy containing 3wt% rhenium and CMSX-10 is third-generation superalloy containing 6–7wt% rhenium. The nominal chemistries for CMSX-4 and CMSX-10 can be found in ^38^ and ^39^, respectively.

CMSX-4 and CMSX-10 Ni-base superalloys contain a two-phase equilibrium microstructure consisting of continuous gamma $\left( \gamma \right)$ matrix and an ordered gamma-prime $(\gamma^{^{\prime}} )$ precipitate. The $\gamma$ is a face-centred-cubic Ni-base austenitic phase and contains a high percentage of solid-solution elements such as Co, Cr, Mo, and W. The $\gamma^{\prime}$ is a coherently precipitating phase generated from the Al, Ti, and Ta content and possesses an $L1_{2}$ cubic crystal structure. The close match in matrix/precipitate lattice parameter $\left( {\sim 0 - 1\% } \right)$ allows the $\gamma^{\prime}$ to precipitate homogeneously throughout the $\gamma$ matrix. Diffraction from the $\gamma^{\prime}$ phase generates both fundamental dips, such as (111), (200), (220), (311), and (400), and superlattice dips, such as (100), (110), (112), and (210), while diffraction from the $\gamma$ phase produces only the fundamental dips ^40^. The $\gamma$ and $\gamma^{\prime}$ lattice parameters for CMSX-4 and CMSX-10 can be found in ^41^ and ^42^, respectively. The alignment of the (001) crystallographic plane with the eventual loading direction, combined with a high-volume fraction of $\gamma^{\prime}$ (up to 70%), and the solid strengthening effects of Cr, W, Ta, and Re, provides single crystal Ni-base superalloys with excellent high-temperature properties.

In this work, CMSX-4 and CMSX-10 bar samples, illustrated in Fig. 1b, were used to investigate bulk (001) plane misorientation perpendicular to the symmetry axis of the casting. CMSX-4 and CMSX-10 disc samples (Fig. 1c) were machined from these bar samples, for the purpose of investigating the influence of a change in the bulk solidification environment (isotherm curvature) on (001) plane misorientation parallel to the withdrawal direction. The CMSX-4 disc sample was taken from the part of the bar closest to the chill plate, the CMSX-10 disc sample the furthest away – please see Fig. 1a for reference.

## Results and discussion

### Bragg-dip mapping of disc sample mosaicity

**Figure 2:**
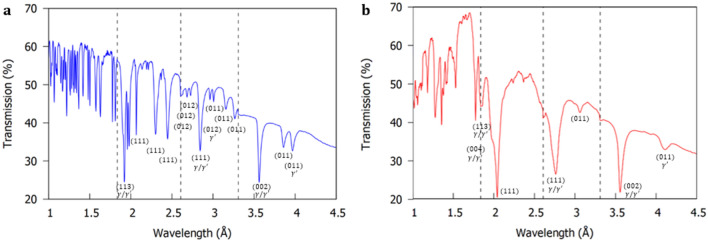
Average neutron transmission spectra for a 2 h 32 min acquisition through the 6 mm thickness of the (**a**) CMSX-4 and (**b**) CMSX-10 disc samples (Fig. 1c). Please see method section for explanation of how the transmission plots are obtained and for the purpose of the vertical dashed lines (experimental shutter positions).

In this work, the disc samples were positioned so that the sample [001] direction (Fig. 1c) was aligned exactly parallel to the incident beam, so that for the (h00) planes $sin\theta$ in Eq. (1) is equal to 1. Both disc samples were separated by 5 mm and positioned completely within the MCP active area (Fig. 9). Figure 2a,b demonstrates the average transmission spectra through the 6 mm thickness of the CMSX-4 and CMSX-10 disc samples (Fig. 1c). The Bragg-dips observed within the transmission spectra were indexed using the procedure outlined in Appendix A of ^43^. For a full explanation of how the transmission plots in Fig. 2a,b are obtained, please see the method section at the end of the article.

**Figure 3:**
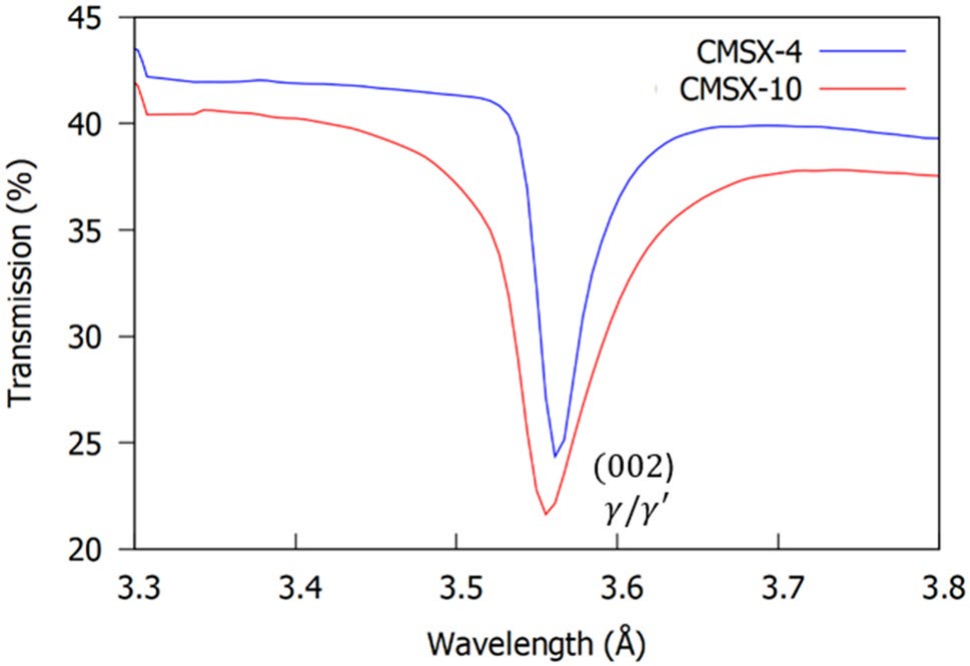
Average neutron transmission spectrum around the (002) convoluted $\gamma /\gamma^{\prime }$ dip. The CMSX-10 Bragg-dip is wider than the CMSX-4 Bragg-dip, indicating a larger distribution of mosaicity within the sample.

From the transmission results in Fig. 2a,b, it is evident most of the Bragg-dips at wavelengths $> 1.6 {{\mathrm{\AA}}}$ are well isolated. In addition, the maximum neutron flux distribution occurs at $2.6 {{\mathrm{\AA}}}$ ^37^. On this basis, the (002) convoluted $\gamma /\gamma^{\prime }$ Bragg-dip (Fig. 3) was chosen for orientation mapping in the disc samples due to the high neutron counts, which decreased noise/error and improved the overall image quality. It is worth mentioning, that in the $< 1.6 {{\mathrm{\AA}}}$ wavelength range the Bragg-dips are highly superimposed (Fig. 2a,b). In this region, the spatial resolution within Bragg-dip mapped images was found to be poor as a result of lower neutron counts, Bragg-dip convolution, and small dip widths.

**Figure 4:**
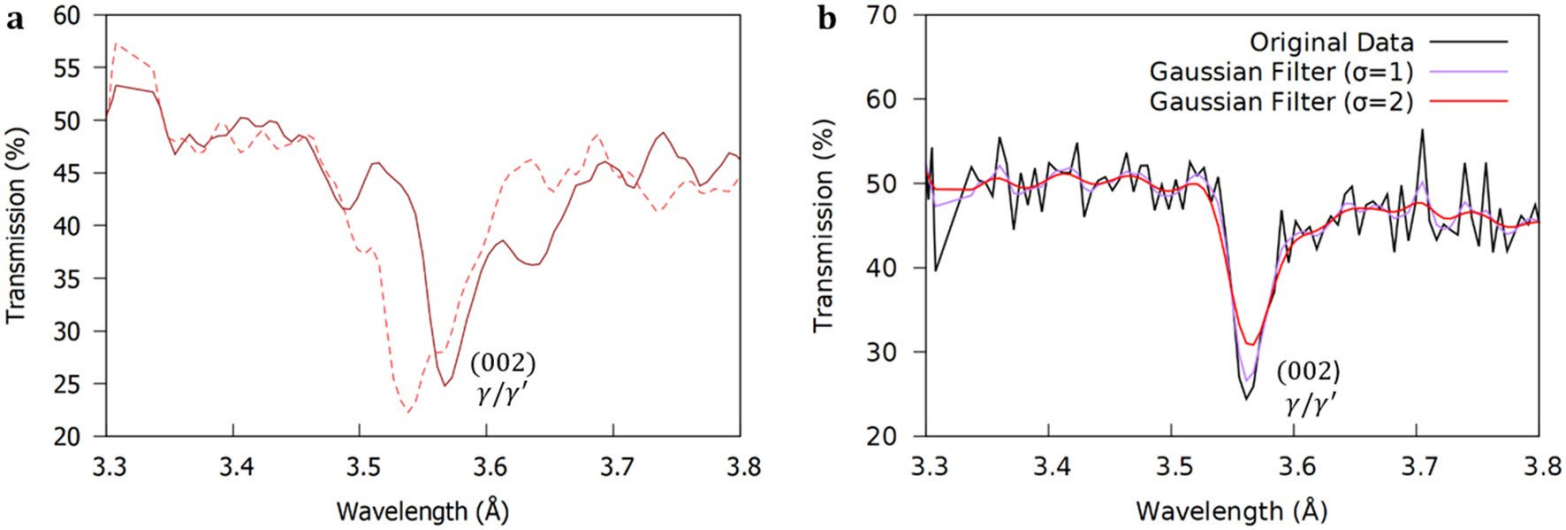
Methodology validation. (**a**) Transmission plot around the convoluted (002) $\gamma /\gamma^{\prime }$ Bragg-dip for two different pixels from Fig. 5b. The lowest transmission wavelength within the (002) Bragg-dip is stored for each pixel. The variation in the value of lowest transmission point between pixels enables construction of a Bragg-dip map of the structure of the averaged sample thickness. (**b**) The influence of a Gaussian filter on the position of the lowest transmission wavelength value for a pixel from the centre of the CMSX-4 disc sample (Fig. 5a). A $\sigma = 1$ was found to negligibly influence the position the lowest transmission wavelength and clearly illuminate the Bragg-dip from the background signal.

**Figure 5:**
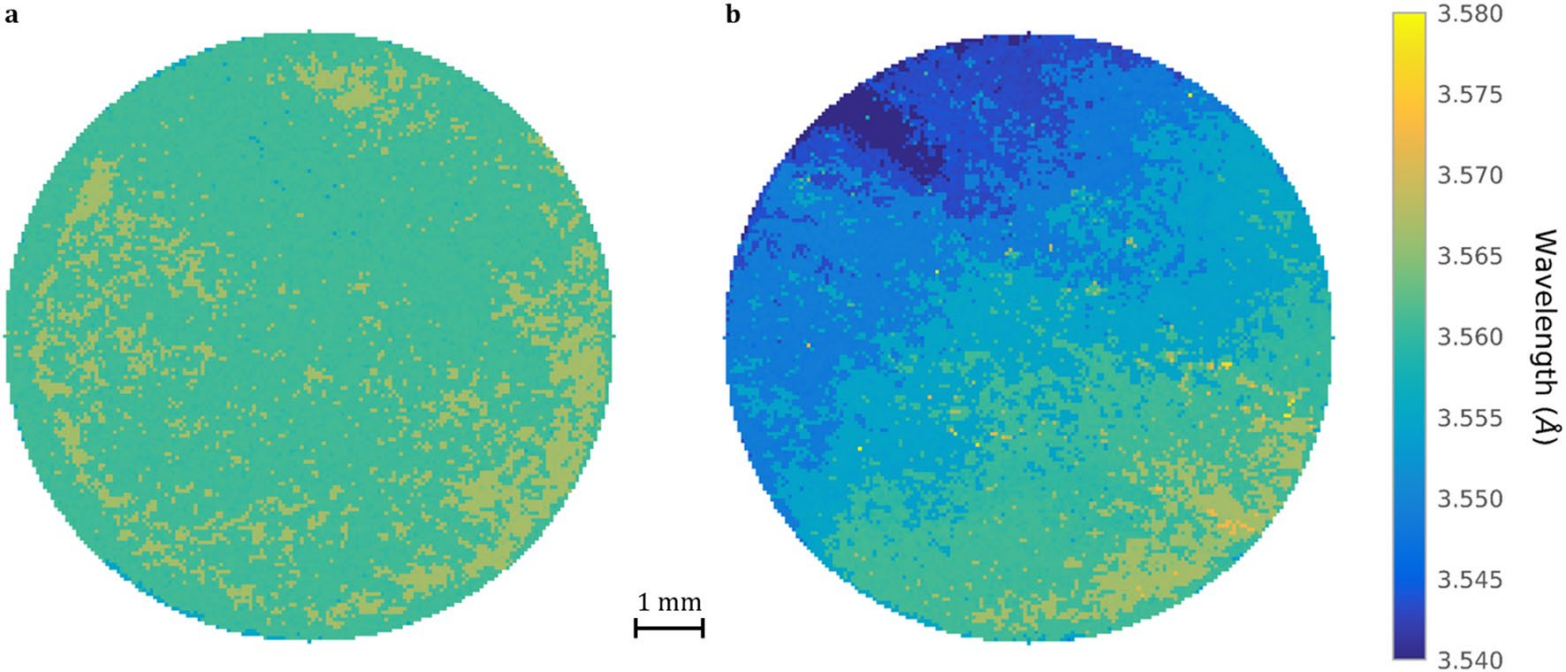
Lowest transmission wavelength Bragg-dip distribution map for the (**a**) CMSX-4 and (**b**) CMSX-10 discs samples of 9.4 mm diameter and 6 mm thickness (Fig. 1c). The images are created by tracking the movement of the lowest transmission wavelength point within the (002) $\gamma /\gamma^{\prime }$ Bragg-dip for each individual pixel. The CMSX-4 sample demonstrates a relatively homogeneous contrast, whereas the CMSX-10 sample possesses a larger colour variation and a banded lowest transmission wavelength profile. Both samples possess a similar maximum lowest transmission wavelength of 3.580 Å, indicated by orange/yellow pixels. (**b**) Possesses the minimum lowest transmission wavelength of 3.540 Å, indicated by dark blue pixels.

To map the convoluted (002) $\gamma /\gamma^{\prime }$ Bragg-dip, the spectrum between $3.3 {-} 3.7 {{\mathrm{\AA}}}$ was extracted from the full wavelength range from each pixel. Following this, a Gaussian filter with $\sigma = 1$ was applied to the spectra to reduce noise and clearly illuminate the $\gamma /\gamma^{\prime }$ Bragg-dip from the background signal (Fig. 4a). From testing, a $\sigma = 1$ was shown to have negligible influence on the position of the lowest transmission wavelength (Fig. 4b). The value of the lowest transmission wavelength was stored for all $512 \times 512$ pixels of the MCP detector and a colourmap created from the range in data (Fig. 5a,b).

Bragg-dip mapping between $3.54 - 3.58 {{\mathrm{\AA}}}$ was found to provide the best image contrast (Fig. 5a,b), where 100% and 97.69% of the lowest transmission wavelengths are within this range, for the CMSX-4 and CMSX-10, respectively. The small percentage of values that fall outside of this range are recorded as bright yellow ($3.58 {{\mathrm{\AA}}}$) or dark blue ($3.54 {{\mathrm{\AA}}}$) pixels (Fig. 5b). Between the two mapped disc images (Fig. 5a,b) there is a clear difference in contrast. The CMSX-10 sample (Fig. 5b) demonstrates a non-homogenous distribution of lowest transmission wavelengths, with the top left of the image possessing the smallest wavelength out of both samples. The CMSX-4 sample (Fig. 5a) displays a relatively homogenous distribution across the entire surface. To quantify the variation in contrast observed between the two mapped images (Fig. 5a,b), the shift in lowest transmission wavelength must be related to an unstrained reference with no mosaicity.

In transmission, the observed width of the Bragg-dip is related to the finite distribution of crystal orientation (mosaicity) and interplanar distances within the single crystal ^28^. Calculating crystal mosaicity for all Bragg-dips within a transmission spectrum is possible using the methods outlined in ^43,44^. However, only the maximum (001) misorientation is of interest within this work, as this is the limiting factor for the creep performance of a single crystal component ^4^. To discuss Fig. 5a,b in terms of mosaicity, requires the microstrain to have minimal influence on the transmission results. Pierret et al*.* ^45^, quantified the maximum microstrain variation in a solutionised Ni-base superalloy turbine blade at $\pm 600$ microstrains. The solutionising process can reduce microstrain by up to ~ 60% ^46^, thus, the maximum observed microstrain within the as-cast disc samples are unlikely to venture over $\pm 1500 \mu \varepsilon$. A value of 1500 microstrains corresponds to a Bragg scattering wavelength shift from an unstrained reference of $\sim 0.0045 {\mathrm{\AA}}$.

In CMSX-4 and CMSX-10 superalloys there is a close match in the matrix/precipitate lattice parameters ^41,42^. In addition, $\gamma^{\prime}$ is the most abundant phase throughout the microstructure $(\sim { }70{{\% }})$. Therefore, the wavelength that corresponds to the average unstrained $\gamma^{\prime}$ crystal lattice spacing, $\overline{\lambda }_{{\gamma_{0}^{\prime } }}$, enables approximation of the maximum wavelength shift in both samples. Using the full width at half maximum (FWHM) of the convoluted (002) Bragg-reflection (Fig. 3), the minimum (and most shifted) wavelength, $\lambda_{min}$, was determined as 3.549 Å and 3.534 Å (Table 1), which corresponds to a maximum wavelength shift of $\sim { }0.03{\mathrm{ {\AA}}}$ and $\sim { }0.04{\mathrm{ {\AA}}}$ (Table 1) for the CMSX-4 and CMSX-10 disc samples, respectively. The observed shift in both samples is almost an order of magnitude larger than that expected for pure microstrain. Thus, the influence of microstrain on the mapped results in Fig. 5a,b is negligible and the shift in wavelengths is almost entirely a result of variation in crystal misorientation. Using the $\lambda_{min}$ values, the maximum misorientation in the CMSX-4 and CMSX-10 disc samples is approximately calculated as $7.6^\circ$ and $8.8^\circ$, respectively. These results are within the allowable (001) misorientation tolerance $\left( {0^\circ - 15^\circ } \right)$ and close to the maximum observed within experiment ^6,47^.

**Table 1:** Measured FWHM data for the average convoluted (002) $\gamma /\gamma^{\prime }$ Bragg-dip for all pixels within the CMSX-4 and CMSX-10 disc samples from Fig. 3; ${\overline{\lambda }}$, ${\uplambda }_{{{\mathrm{min}}}}$, ${\uplambda }_{{{\mathrm{max}}}}$ are the average, minimum, and maximum wavelength values, respectively. $\overline{\lambda }_{{\gamma_{0}^{\prime } }}$ corresponds to the average unstrained wavelength for the $\gamma^{\prime }$ phase; $\sigma_{{\lambda_{{\gamma_{0}^{\prime } }} }}$ the standard deviation of measured unstrained $\gamma^{\prime }$ wavelengths.

Material	$\overline{\lambda }_{{\gamma_{0}^{\prime } }}$(Å)	$\sigma_{{\lambda_{{\gamma_{0}^{\prime } }} }}$(Å)	FWHM (Å)	$\overline{\lambda }$(Å)	$\lambda_{min}$(Å)	$\lambda_{max}$(Å)	$\theta_{max}$(Å)
CMSX-4	3.580 ^41^	0.003 ^41^	0.0296	3.564	3.549	3.579	7.6
CMSX-10	3.576 ^42^	0.002 ^42^	0.0510	3.559	3.534	3.585	8.8

Within Fig. 5a,b, regions where $\lambda \cong \overline{\lambda }_{{\gamma_{0}^{\prime } }}$ indicate locations where the (001) crystallographic plane is more well aligned and parallel with the neutron beam throughout the entire sample thickness; identified by orange/yellow pixels. It follows, that if any (001) plane misorientation exists, the lowest transmission wavelength when viewed parallel to the neutron beam will decrease. It is clear from Table 1, that CMSX-4 has a smaller FWHM than the CMSX-10 sample, thus, there is less variation in (001) plane misorientation across the microstructure. This is visualised in Fig. 5a as a relatively homogeneous distribution of contrast throughout the mapped image. Therefore, the bulk axial thermal gradient was strong and relatively uniform across the sample at the time of solidification; characteristic of solidification close to the chill plate (Fig. 1a). It is important to note, that this sample still possesses some bulk misorientation as $\overline{\lambda } \ne \overline{\lambda }_{{\gamma_{0}^{\prime } }}$ (Table 1).

On the other hand, the CMSX-10 sample demonstrates a much larger FWHM (Table 1). In this sample, only the lower right-hand side of the mapped image (Fig. 5b) obtained a $\lambda \cong \overline{\lambda }_{{\gamma_{0}^{\prime } }}$. Therefore, only at this location is the (001) plane well orientated and almost parallel with the neutron beam. Consequently, only this region formed under a relatively flat solid/liquid isotherm. Another important result is the location of the largest and most detrimental (001) plane misorientation, which occurs in a small and isolated part of the sample in the top left, on exactly the opposite side to the well orientated (001) plane (Fig. 5b). This variation in (001) orientation across the bulk microstructure is characteristic of solidification under a curved isotherm ^48^. Interestingly, the severity of (001) plane misorientation increases across the sample in defined texture bands, which have formed perpendicularly along the line of greatest (001) misorientation. These texture bands are a few mm thick and possess some inherent waviness (Fig. 5b). It follows, that solidification under a curved isotherm will induce a non-uniform stress distribution across the bulk microstructure. As misorientations are known to be a result of thermomechanical deformations ^5^, the thickness of the texture bands must be directly related to: (1) the local thermal contraction bending stress; (2) the shape of the solid/liquid isotherm. This new texture band phenomenon may be significant in determining the origin behind high angle grain boundary defects within turbine blades ^49^ and quantifying the mechanism behind single crystal mosaicity.

### Bragg-dip mapping of bar sample mosaicity

**Figure 6:**
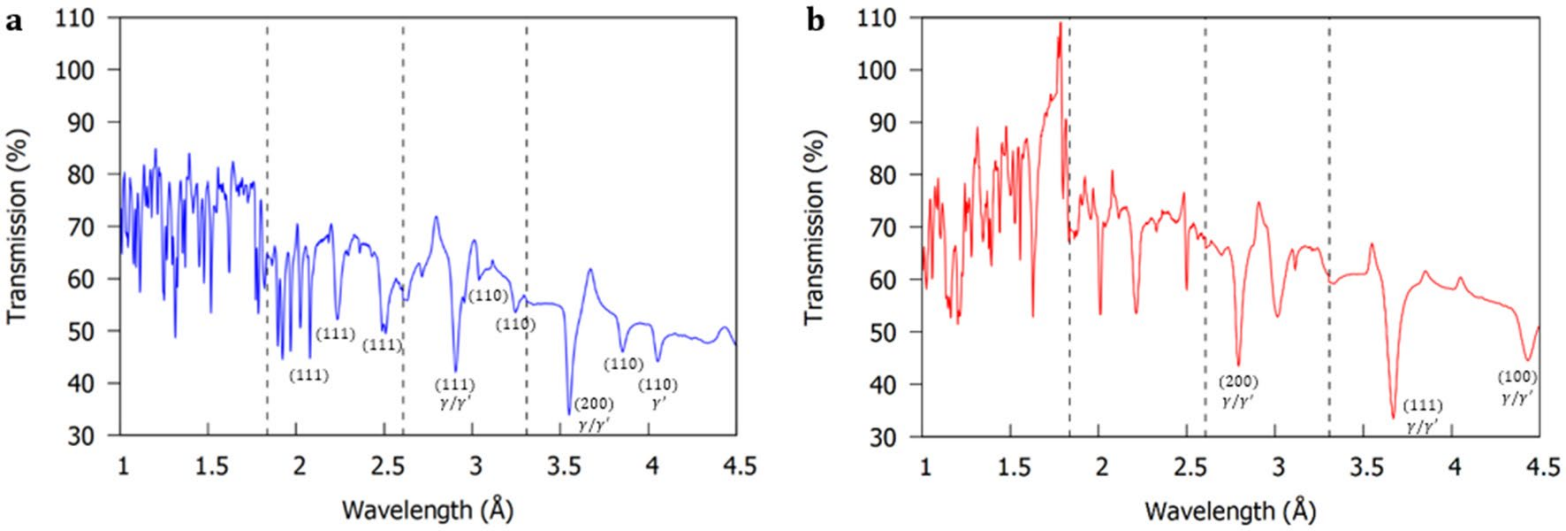
Average neutron transmission spectra for a 3 h 13 min acquisition for the (**a**) CMSX-4 and (**b**) CMSX-10 bar samples. Please see method section for explanation of how the transmission plots are obtained and for the purpose of the vertical dashed lines (experimental shutter positions).

In this work, both bar samples (Fig. 1b) were orientated so that their long axis were aligned exactly perpendicular to the incident beam. They were positioned 5 mm apart and within the centre of the MCP active area ($28 \times 28{ }\,{\mathrm{mm}}^{2}$), therefore, leaving 18 mm of the top and bottom of both samples outside of the MCP field of view. The resultant Bragg-dip transmission spectra through the averaged thickness of the CMSX-4 and CMSX-10 bar samples are demonstrated in Fig. 6a,b. The Bragg-dips observed within the transmission spectra were indexed using the procedure outlined in Appendix A of ^43^. For a full explanation of how the transmission plots are obtained in Fig. 6a,b, please see the method section at the end of the article.

**Figure 7:**
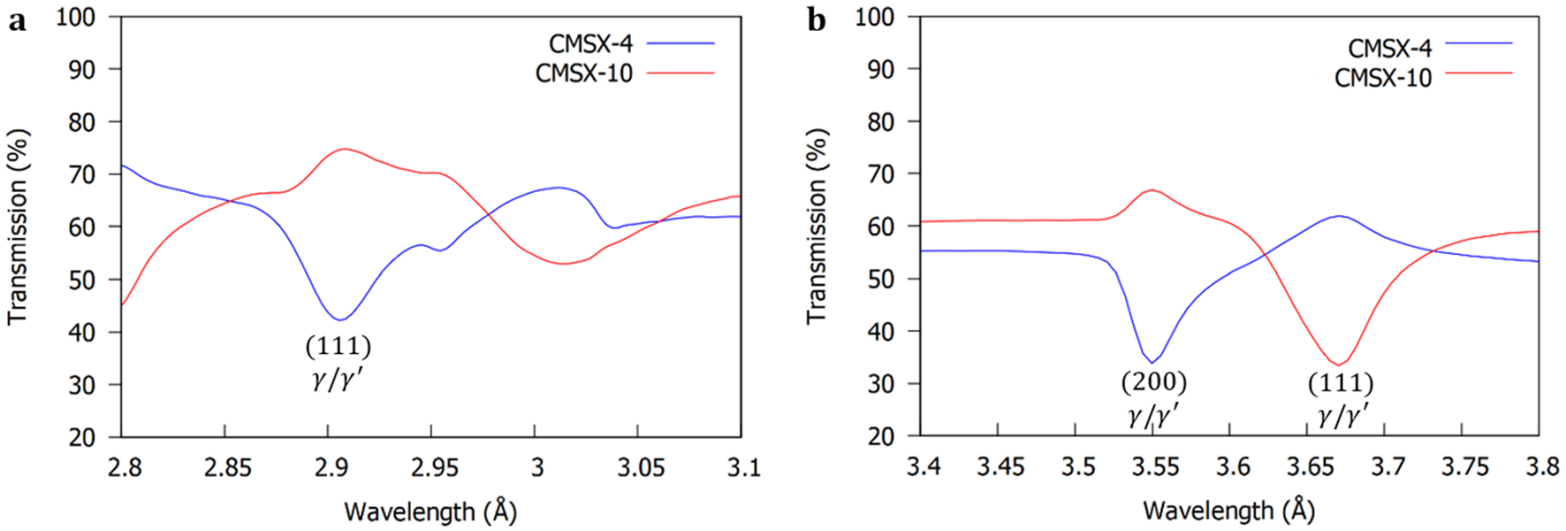
Average neutron transmission spectra for the CMSX-10 and CMSX-4 bar samples at (**a**) the Bragg-dips that provided the best image contrast in Fig. 8a,b and (**b**) the convoluted (200) and (111) $\gamma /\gamma^{\prime }$ Bragg-dips that clearly demonstrated the neutron scattering effect. Diffraction occurs at different wavelengths in each sample due to the crystallographic directions not being aligned identically.

Before Bragg-dip mapping of the bar samples is attempted, it is important to discuss an interesting trend observed in Fig. 6a,b. A dip in the recorded transmission of one plot results in a corresponding jump in transmission within the other. In fact, in Fig. 6b, the transmission goes over 100% at $\sim 1.7 {{\mathrm{\AA}}}$. This phenomenon is clearly highlighted in the transmission versus wavelength relationship in Fig. 7a,b. In both disc and bar tests the samples were separated by 5 mm (Fig. 9), however, this jump is only clearly observed in the transmission spectra of the bar samples (Fig. 6a,b). In the bar experiment, the furthest part of both samples was positioned an additional 6 mm further from the MCP detector than the discs (see method section), thus, increasing beam divergence. Consequently, the observed peaks within the transmission spectra of the bar samples (Fig. 6a,b) occur as a result of neutrons from one sample scattering to the detector in the area corresponding to the position of the other sample, adding neutron counts.Therefore, when performing mosaicity mapping, it is suggested to minimise the furthest distance from sample to detector and/or increase the specimen separation to reduce noise, thereby increasing mapped spatial resolution.

**Figure 8:**
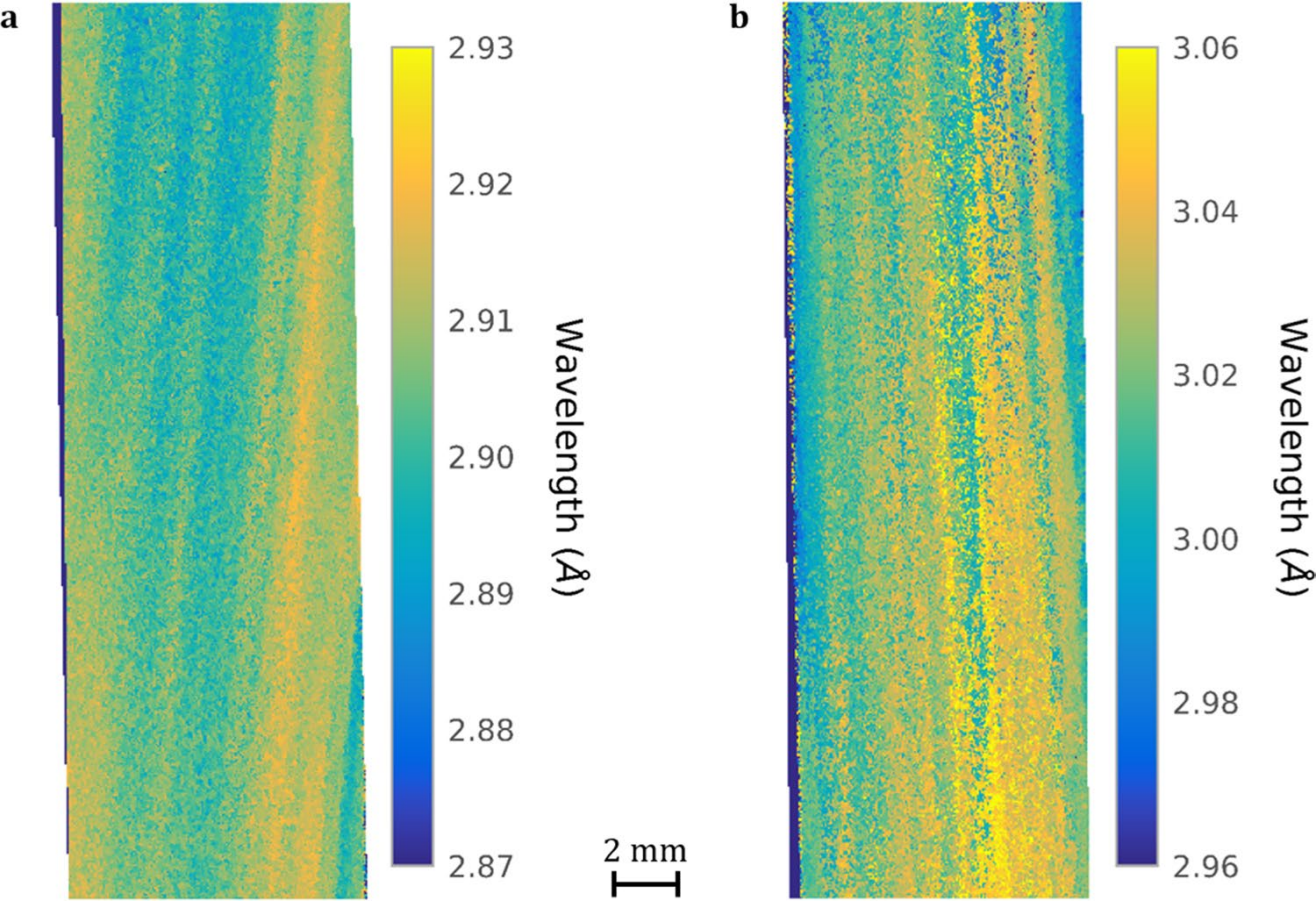
Lowest transmission wavelength Bragg-dip distribution map for the longitudinal (**a**) CMSX-4 and (**b**) CMSX-10 bar samples of 9.4 mm diameter and 28 mm height (max MCP active area). Both mosaicity maps are acquired from the centre of each bar. The images are created by tracking the movement of the lowest transmission wavelength for each pixel between 2.87–2.93 Å and 2.96–3.06 Å for CMSX-4 and CMSX-10, respectively. The bottom of both samples solidified closer to the chill plate than the top. The visibility of the (001) plane misorientation increases with height from the chill plate.

**Figure 9:**
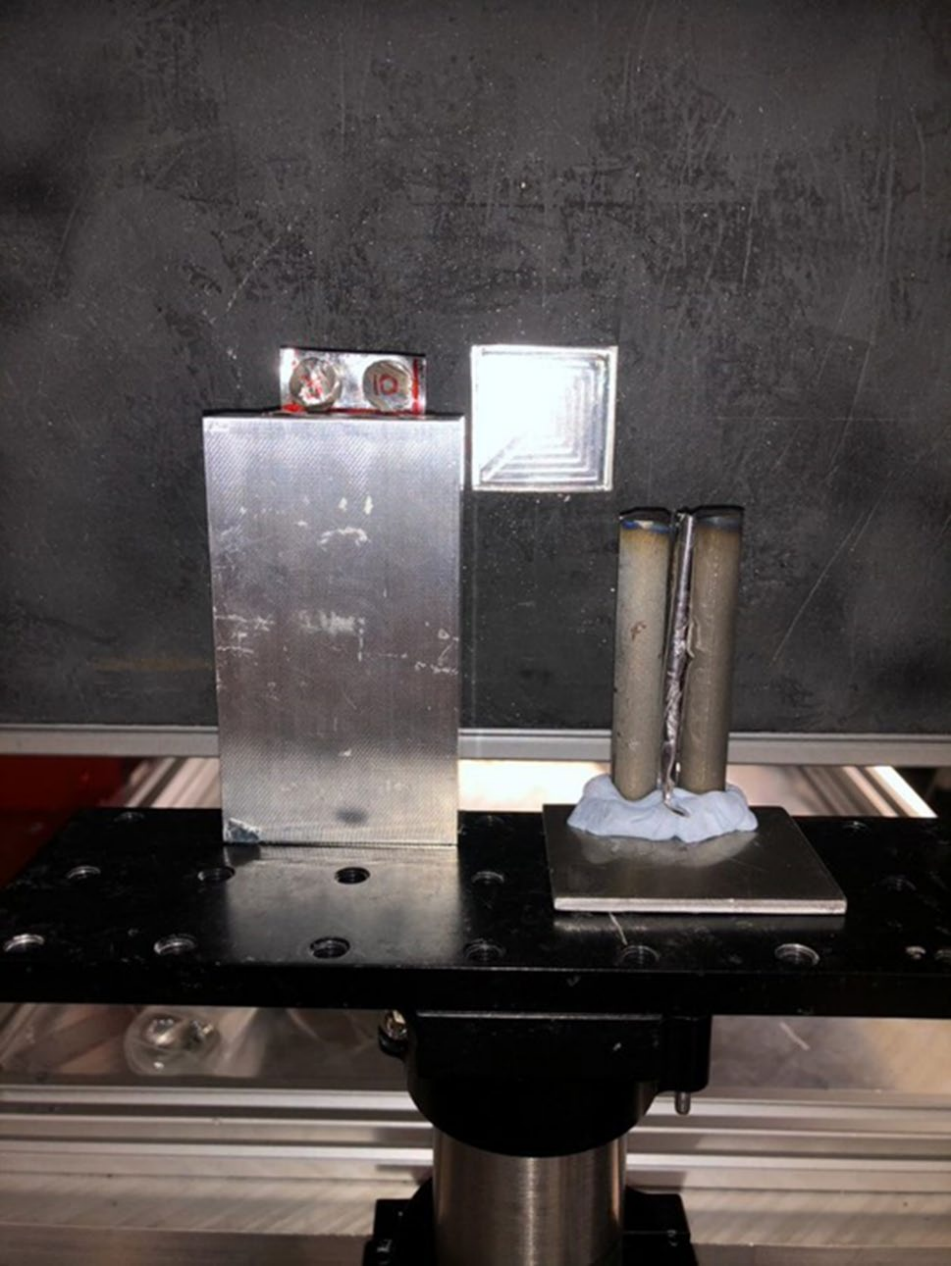
Image of the experimental set-up. The small disc samples are positioned on the left-hand side and 13 mm in front of the MCP detector active area. The discs are 6 mm thick and 9.4 mm in ∅. The bar samples are positioned on the right-hand side and 19 mm in front of the MCP detector active area. The bars are 9.4 mm in ∅ and 64 mm in height. Only the centre 28 mm of the bars are imaged (maximum MCP field of view). The samples are positioned 56 m from the neutron moderator and sample separation is 5 mm.

To decrease this scattering effect and improve overall image quality, non-overlapping Bragg-dips were chosen. Below $\sim 2 {\mathrm{\AA}}$ (Fig. 6a,b), the image resolution was found to be quite poor and the Bragg-dip width small, which provided insufficient contrast across the entire height of the sample. From trial and error, the convoluted (111) $\gamma$/$\gamma^{\prime}$ Bragg-dip at 2.9 Å (Fig. 7a) for the CMSX-4 sample was found to develop the best image contrast. For the CMSX-10 sample, the best contrast was determined at the 3.02 Å Bragg-dip. This dip corresponds to either the convoluted (111) or (200) $\gamma$/$\gamma^{\prime}$ plane reflections. However, because the incident beam was not closely aligned with the [010] crystallographic direction within this sample, it is not possible to accurately index the 3.02 Å Bragg-dip in the present work; a detailed follow up study will be undertaken in due course.

The lowest transmission wavelength for each pixel between $2.87{ }{-}{ }2.93{\mathrm{ {\AA}}}$ and $2.96{ }{-}{ }3.06{\mathrm{ {\AA}}}$ was used to map the CMSX-4 (Fig. 8a) and CMSX-10 (Fig. 8b) samples, respectively. This wavelength range is an order of magnitude larger than that required for the disc sample mapping in Fig. 5a,b. Therefore, the mapped bar samples are almost pure representations of inherent texture variation through the averaged thickness. The chosen Bragg-dips in Fig. 7a are characterised by a large drop in transmission and produce clearly defined axial misorientations of the (001) plane in respect to sample [001] direction (Fig. 8a,b). It is clear from Fig. 8a,b that both samples possess some inherent spiral rotation from the bottom to top of the image. In Fig. 8a this twisting is visualised as an anticlockwise rotation about the sample long axes, whereas in Fig. 8b there is an apparent clockwise rotation. It should be noted, that the relative lattice misalignment can be quantified from the shifting of the Bragg-dips from an unstrained reference, however, crystallographic rotation with respect to [001] cannot be obtained due to lack of reference. Interestingly, for a spiral rotation of the (001) plane to exist, the bulk heat extraction direction must be changing with height from the chill plate. A 3D reconstruction of bulk single crystal mosaicity would enable quantification of this new spiral rotation phenomenon, which is the focus of a detailed follow up study.

At the bottom of both images (Fig. 8a,b) there is less texture variation, the same trend is observed in the disc sample in Fig. 5a. This occurs due to the high axial thermal gradient that exists close to the chill plate (Fig. 1a), which results in microstructural refinement ^50^. Unfortunately, geometric resolution in this work was limited to $\sim 100 \upmu \text{m}$ (see method section for calculation), which makes distinguishing small variations in texture difficult once the channel widths become finer. With regard to the previous discussion, if single crystal mosaicity was a local phenomenon i.e., randomly distributed, then the Bragg-dip intensity throughout the sample thickness would be uniform and provide no image contrast. However, the clear visualisation of (001) plane axial misorientation (Fig. 8a,b) indicates that mosaicity is a macroscale phenomenon that occurs in defined channels, which is consistent with the texture bands observed in Fig. 5b.

## Conclusions

Single crystal mosaicity has been successfully mapped on the IMAT beamline using time-of-flight energy-resolved neutron imaging. The novel Bragg-dip mapping technique has enabled visualisation and quantification of bulk (001) crystallographic plane misorientation in respect to the sample [001] direction. Furthermore, the relationship between bulk mosaicity and variations in the bulk solidification environment has been elucidated. As a result, a new spiral twisting solidification phenomena has been observed, which is theorised to be related to changes in the isotherm shape with height from the chill plate. This novel Bragg-dip mapping methodology enables post-mortem deduction of the 3D solid/liquid isotherm shape through the relationship between (001) misorientation and the bulk heat flow direction. This is important, as a quantitative representation of solid/liquid isotherm curvature enables direct comparison with investment casting software such as ProCAST (ESI Group, Paris, France) for validation of binary and multicomponent alloy modelling. This new Bragg-dip mapping methodology provides the foundation for combining multiple 2D Bragg-dip mapped images, into a full 3D reconstruction; ideal for quantifying bulk mosaicity, channel ordering, and spiral rotation within the microstructure. The cold neutron imaging and diffraction instrument, IMAT, enables precise quantification and investigation into single crystal mosaicity as a function of variations in the solidification environment. Consequently, IMAT will be established as a fundamental characterisation tool for material structure and property analysis in the future.

## Methods

### Determination of the experimental parameters for the Ni-base superalloy samples

Neutron TOF spectrum were recorded over a range of $1.51 {-} 97.1 \,\mathrm{ms}$ and a wavelength range of $0.1 {-} 6.8 {{\mathrm{\AA}}}.$ The collimation ratio, $L_{p} /D$, where $L_{p}$ is the distance between pinhole and sample ($L_{p} = 10 m$) and $D$ is the pinhole size ($D = 60 mm$), defined the local divergence of the neutron beam to maintain sufficient flux at the sample. The maximum obtainable geometric resolution, $U_{g}$, given by Eq. (3):

3 $$U_{g} = \frac{x}{{L_{p} /D}},$$

where, $x$ is the furthest distance from the sample to the detector and should be minimised to reduce divergence of the neutron beam to achieve the greatest resolution. The disc and bar samples were positioned 13 mm and 19 mm from the active area of the MCP detector, which provided an ultimate geometric resolution of 78 μm and 114 μm, respectively.

Both sets of samples were separated by 5 mm and placed in front of the MCP detector active area. The IMAT sample positioning system (SPS) was used for the sample alignment. The $x$, $y$, and $z$ directions of the SPS in the home position are aligned with the beam direction, transverse direction, and vertical direction, respectively. The samples were visually aligned using the alignment laser and the SPS controls and then fine-tuned in-situ in the neutron beam using the PIXELMAN ^51^ GUI of the MCP as a live display.

### Calculation of sample transmission using time-of-flight data

During image acquisition, recorded neutron fluxes from the sample contain not only sample statistics but also beam and detector properties. Therefore, to determine the transmission image, neutron beam data with and without the sample is required to improve image quality and establish a baseline. The open beam, also known as flat field correction, eliminates the effects of non-uniformity in the incident beam and non-uniform response of the detection system from the radiographs. The MCP settings were the same for the sample and the open beam runs. The open beam collection time was the same as sample measurement time.

The data acquisition was controlled by a python script which includes the sample coordinates, exposure time (in proton current), data folder and data filename definitions; for a full explanation of the IMAT data collection process the reader is referred to ^37^. Utilising a target proton current for data acquisition ensures effective sample exposure time in case the neutron source trips and/or neutron pulses are vetoed by, for example, the chopper system or sample environment ^37^. The total proton current for the disc and bar samples was 100.0059 μA and $127.7611{ }\upmu \text{A}$ which was equivalent to $2h 31m 44s$ and $3h 13m 1s$ acquisition time, respectively. The proton current/acquisition time for the bars was increased due to an increase in sample thickness of 3.4 mm. A stack of 2711 radiographies were collected for each test, with each radiography belonging to a specific wavelength.

The data was corrected prior to Bragg-dip analysis following a method used by the BEAn software ^52^. Six MCP readouts (shutter positions) were chosen to reduce the event overlap ^53^. Event overlap occurs due to artefacts (dip in transmission) as a result of the probability of neutron capture falling as neutrons are registered (between readouts). Thus, the efficiency at which neutrons are captured decreases with the number of neutrons captured. To account for this dip in transmission, Poisson statistics were used to calculate the actual events that should have been detected between readouts.

The open beam and sample beam data after overlap correction were scaled to the same number of incident neutrons. This was achieved by reducing the open beam data down to the same number of counts using a multiplication factor as described in ^52^. Once scaled the sample stack was normalised by dividing pixel by pixel by the open beam data to calculate a stack of transmission images. Both open beam and sample stacks are then subjected to artefact cleaning by filling the dead pixels using averages of surrounding neighbours.

## Data Availability

The datasets generated during and/or analysed during the current study are available at https://doi.org/10.5286/ISIS.E.RB1955012.
